# Supplementary material for: Enhanced validation of antibodies for research applications
Source: Nat Commun. 2018 Oct 8;9:4130. doi: 10.1038/s41467-018-06642-y (PMC6175901; doi:10.1038/s41467-018-06642-y)
Supplement: Supplementary file 1 — Description of Additional Supplementary Files [file 41467_2018_6642_MOESM1_ESM.docx]

**Title:** Supplementary Data 1

**Description:** List of the 6,014 antibodies evaluated in this study

**Title:** Supplementary Data 2

**Description:** TPM values for all genes in 56 cell lines

**Title:** Supplementary Data 3

**Description:** Relative protein abundance (normalized reporter intensity) determined by TMT10plex

**Title:** Supplementary Data 4

**Description:** Ratio to standard determined by PRM

**Title:** Supplementary Data 5

**Description:** Western Blot band intensities used for orthogonal validation

**Title:** Supplementary Data 6

**Description:** Antibodies validated within HPA

**Title:** Supplementary Data 7

**Description:** Capture MS intensities for each analyzed gel piece

**Title:** Supplementary Data 8

**Description:** Molecular weight for the largest transcript

**Title:** Supplementary Data 9

**Description:** Protein quantification by TMT proteomics performed in triplicate

**Title:** Supplementary Data 10

**Description:** PRM peptide coordinates

**Title:** Supplementary Data 1

**Description:** Peptide ratios from PRM used to determine protein abundance
