# Supplementary material for: Enhanced validation of antibodies for research applications
Source: Nat Commun. 2018 Oct 8;9:4130. doi: 10.1038/s41467-018-06642-y (PMC6175901; doi:10.1038/s41467-018-06642-y)
Supplement: Supplementary file 2 — Supplementary Information [file 41467_2018_6642_MOESM2_ESM.pdf]

Enhanced validation of antibodies for research applications

Edfors *et al.*

Supplementary Information

File	Description
Supplementary Figure 1	Expression profile of cell line panel used for Western blot validation
Supplementary Figure 2	Western blot images and TMT validation including loading control and
Supplementary Figure 3	Western blot images and PRM validation including loading control
Supplementary Figure 4	Scatter plots of PRM and TMT orthogonal validation
Supplementary Figure 5	Orthogonal validation using a standardized assay
Supplementary Figure 6	Examples of independent antibody validation
Supplementary Figure 7	Examples of genetic validation (i.e. siRNA knockdown)
Supplementary Figure 8	Examples of recombinant expression validation
Supplementary Figure 9	PSMA1 as loading control for Western blot application
Supplementary Figure 10	SDS-PAGE gel total protein image used for capture MS validation
Supplementary Table 1	The cell lines used in this study
Supplementary Table 2	Summary of genes quantified by proteomics-based methods
Supplementary Table 3	Pearson's r and Spearman rho estimates for all Western blot bands and their correlation with MS and RNA data
Supplementary Table 4	Criteria for validation
Supplementary Table 5	Success rate per method
Supplementary Table 6	QPrESTs used in this assay
Supplementary Data 1	List of the 6,014 antibodies evaluated in this study
Supplementary Data 2	TPM values for all genes in 56 cell lines
Supplementary Data 3	Relative protein abundance (normalized reporter intensity) determined by TMT10plex
Supplementary Data 4	Ratio to standard determined by PRM
Supplementary Data 5	Western Blot band intensities used for orthogonal validation
Supplementary Data 6	Antibodies validated within HPA
Supplementary Data 7	Capture MS intensities for each analyzed gel piece
Supplementary Data 8	Molecular weight for the largest transcript
Supplementary Data 9	Protein quantification by TMT proteomics performed in triplicate
Supplementary Data 10	PRM peptide coordinates
Supplementary Data 11	Peptide ratios from PRM used to determine protein abundance

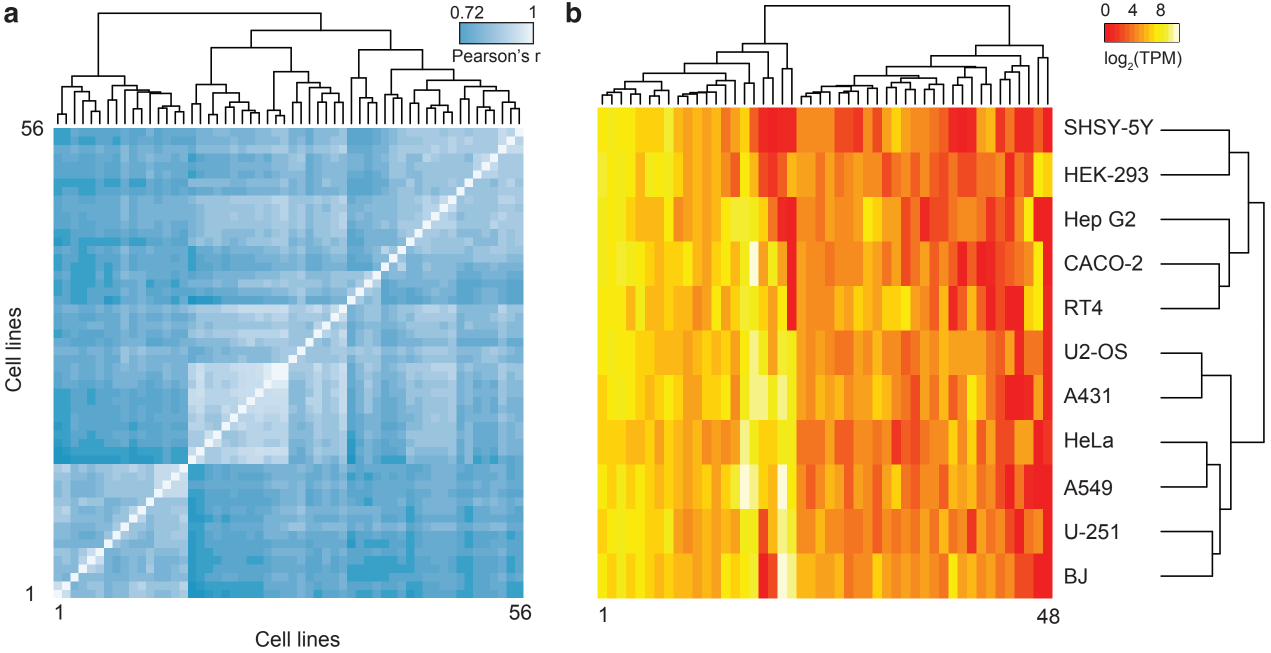

Figure S1 - Transcriptomics-based selection of cell-line panel used for orthogonal validation of antibodies for Western blot application. a. Clustering of cell lines based on correlation given as Pearson's R for the gene expression from 56 human cell-lines. Expression given as Transcript Per Million (TPM) was log_2 transformed and we included all protein coding genes covered by the Human Protein Atlas (n=19,628) in the analysis. Cell lines were chosen based to be as distant from each other as possible in the dendrogram. The transcriptomics data for all cell lines can be found in **Table S3**. **b.** Gene expression (log_2(TPM)) across cell lines for the 48 genes used in the orthogonal validation.

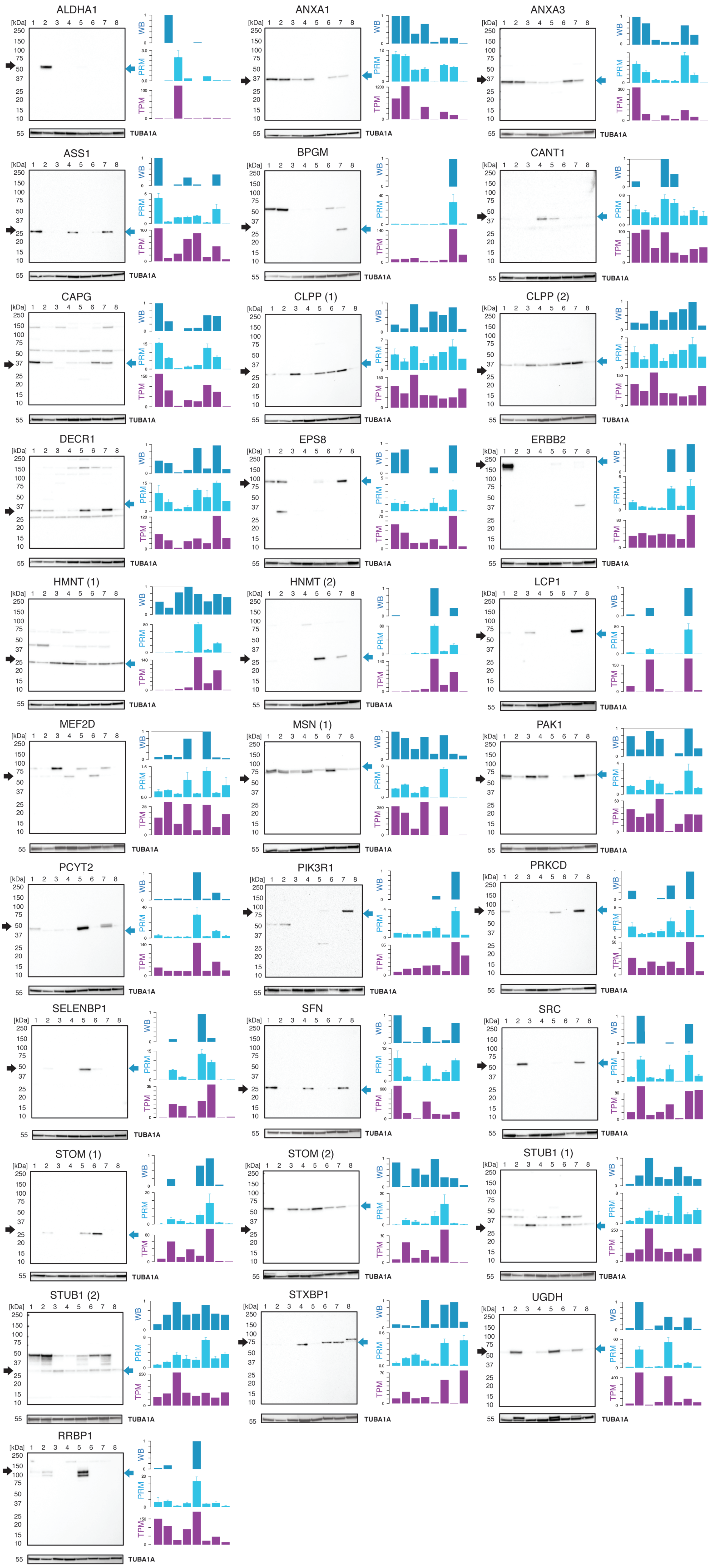

Supplementary Fig. 2 - Western blots for antibodies and corresponding protein levels quantified by PRM and RNA-levels determined by RNA-seq. The assay was performed on eight cell-lines (A431, A549, HEK-293, U-2 OS, Hep G2, HeLa, RT4 and SH-SY5Y). The black arrow indicates expected molecular weight, and the blue arrow represents the integrated Western blot band used in the analysis. A loading control is included for all blots targeting tubulin 1-alpha (TUBA1A). Western blot intensities (WB) are summarized in **Supplementary Table 16**. Ratio-tostandard (PRM) are summarized in **Supplementar Table 4** and Transcripts per million (TPM) are summarized in **Supplementar Table 3.** Error bars represent 1 s.d. across three technical replicates.

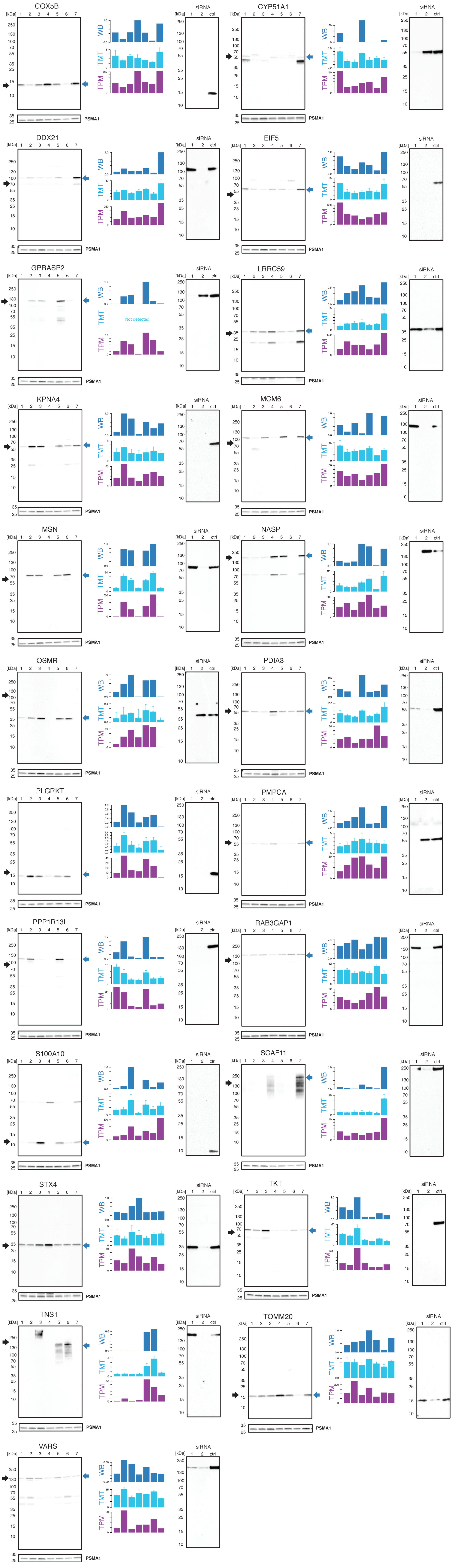

Supplementary Fig. 3 - Western blots for antibodies and complementary protein levels quantified by TMT and RNA-levels determined by RNA-seq for seven cell-lines (RT4, U-251, A549, Hep G2, U-2 OS, BJ, CACO-2). The black arrows indicate expected molecular weight, and the blue arrows represent the integrated Western blot band used in the analysis. A loading control is included for all blots targeting the proteasome subunit alpha 1 (PSMA1, Figure S7). Each antibody were verified by siRNA knock-down in U-2 OS using two siRNA reagents (1-2), including one scrambled siRNA as control (ctrl). Western blot intensities (WB) are summarized in **Supplementary Table 16**. Normalized reporter ion intensiteis (TMT) are summarized in **Supplementary Table 5** and Transcripts per million (TPM) are summarized in **Supplementary Table 3**. Error bars represent 1 s.d. across three technical replicates.

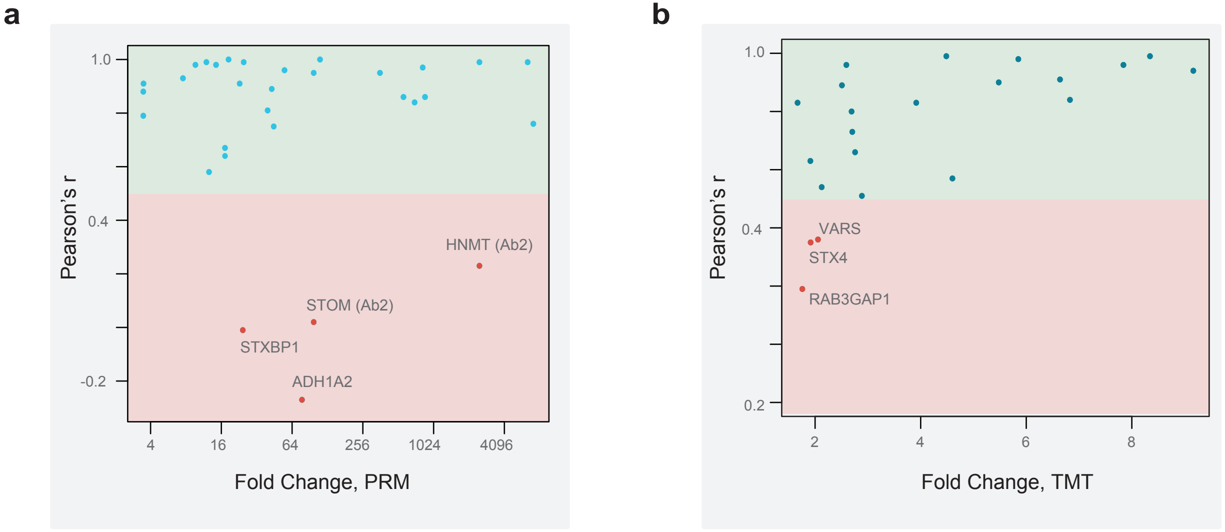

Figure S4 – Scatter plots of fold change for PRM and TMT. a. Scatter plot showing the correlation as a function of difference in protein levels determined by PRM. **b.** Scatter plot showing the correlation as a function of difference in protein levels determined by TMT

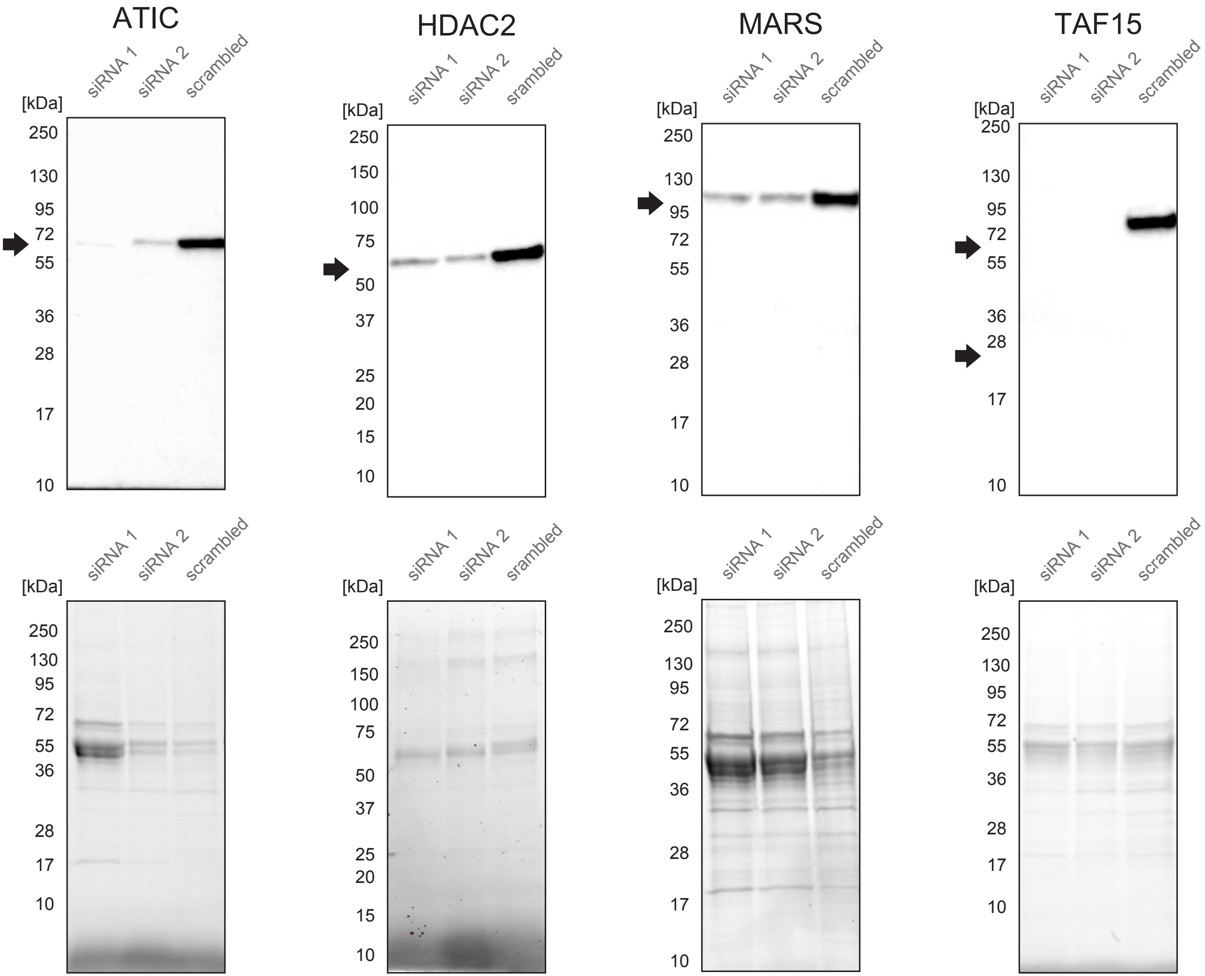

Supplementary Figure 7 - Examples of genetic validation (i.e. siRNA knock-down). Four example genes with antibodies validated by siRNA knock-down. The top row shows two target specific siRNA reagents together with a control (scrambled) for each antibody. The black arrow represents expected molecular weight. The lower row represents total protein gel images used as loading control for each Western blot.

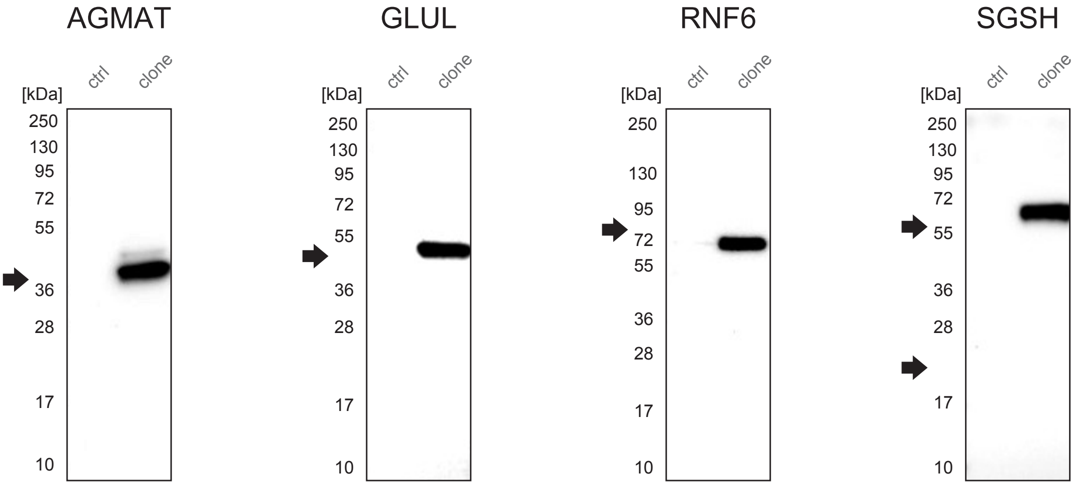

Supplementary Figure 8 - Examples of recombinant expression for antibody validation. Four example genes with overexpression lysates (clone) of the protein target including non-ex-pressing control (ctrl). The black arrow represents expected molecular weight.

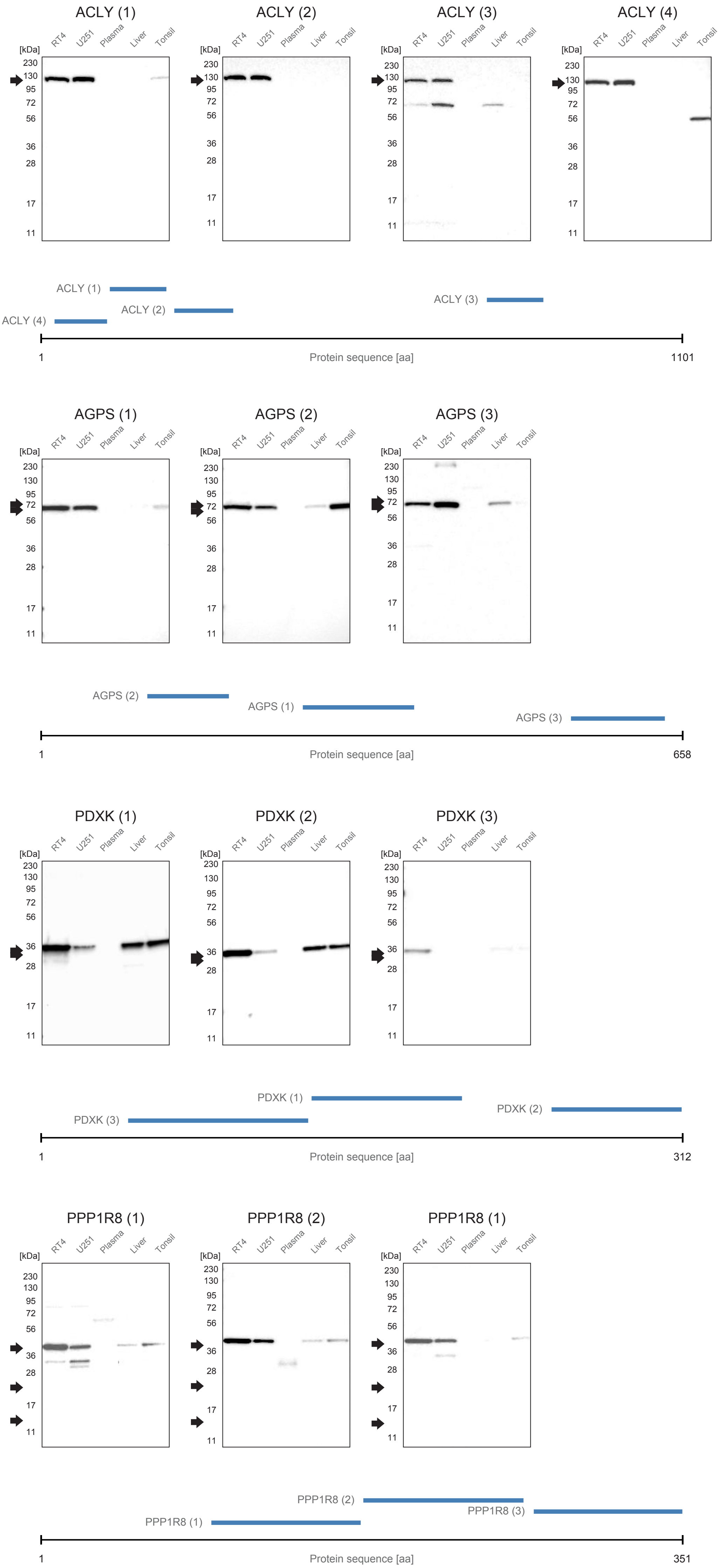

Supplementary Figure 6 - Examples of independent antibody validation. Four example genes with non-overlapping antigen sequences used for independent antibody validation performed in a panel consisting of two cell-lines (RT4, U-251), human plasma, and two tissues (liver, tonsil). The black arrow represents expected molecular weight, and the schematic figure illustrates protein fragment sequences (blue) used for antibody generation

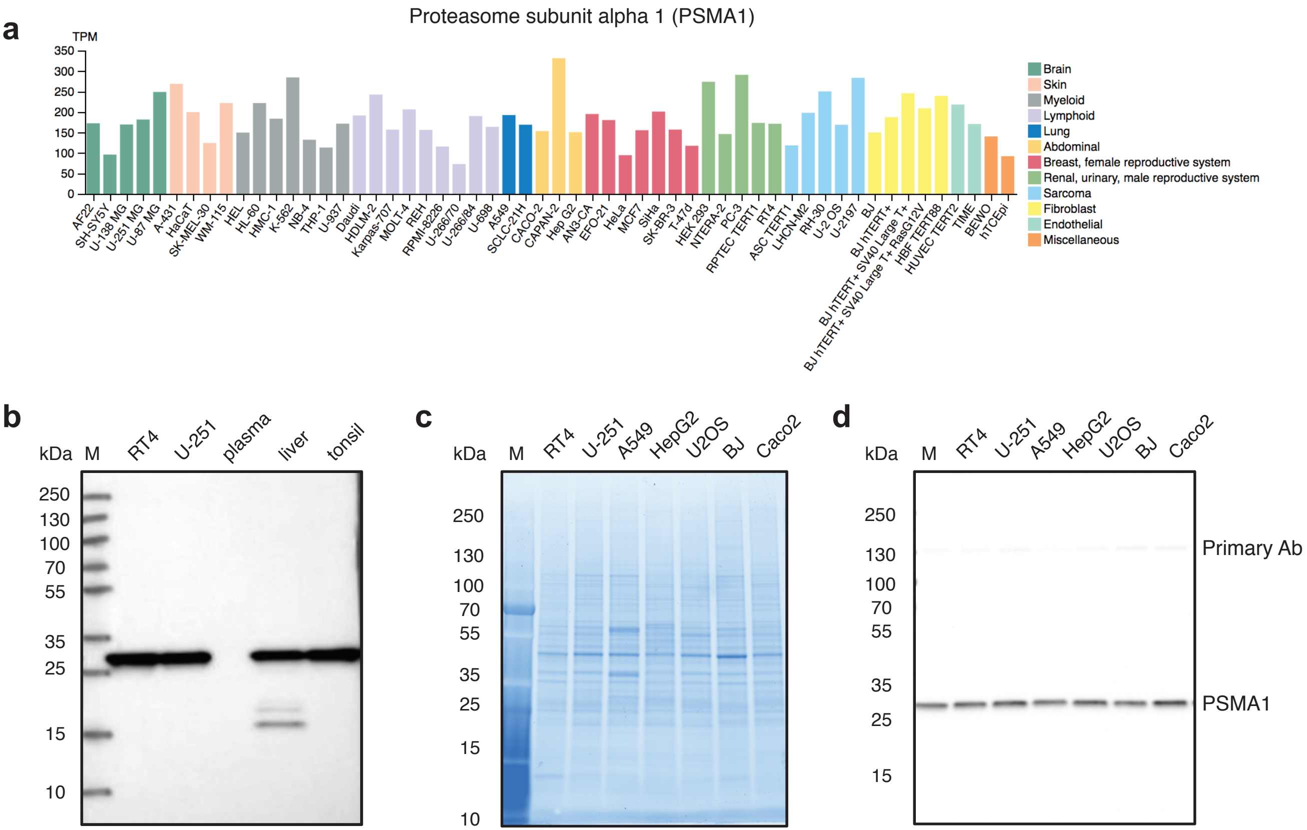

Supplementary Figure 9 - Selection criteria for anti-PSMA1 as loading control for Western blot analysis across different cell lines. a. Transcriptomics analysis of PSMA1 transcripts across 56 cell lines. **b.** Original Western blot from high throughput Western blot analysis performed within the Human Protein Atlas project (HPA037646). **c.** SDS-PAGE analysis of total protein content for seven cell-lines used for Western blot analysis for 24 antibodies. d. HPA037646 anti-PSMA) as loading control and co-exposed with Primary Ab, (anti-RAB3GAP1).

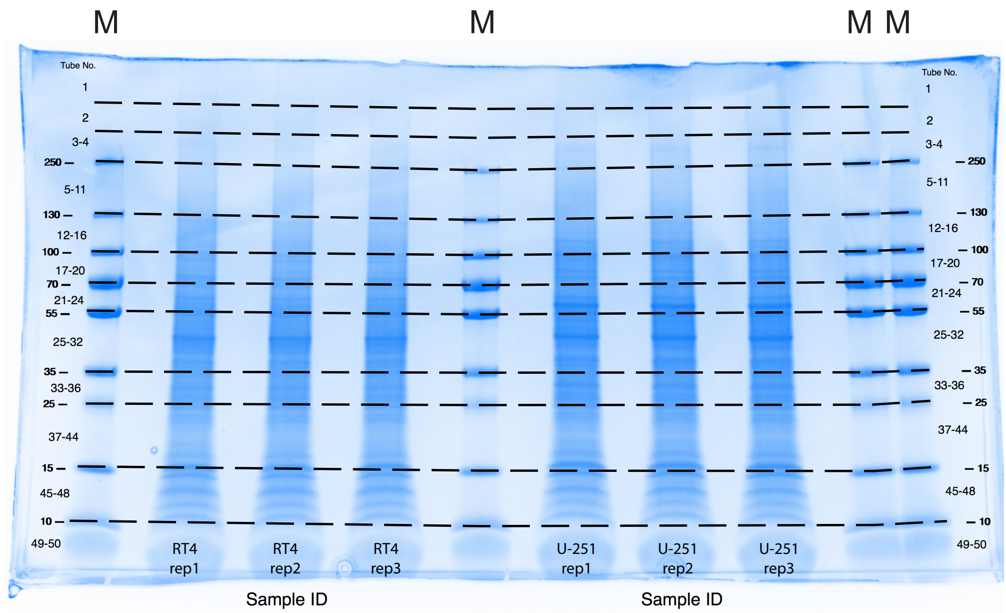

Supplementary Figure 10 - SDS-PAGE gel cutout. Bold numbers represent the molecular weight of the protein ladder. Each lane was cut into 50 individual gel-pieces (1-50), containing proteins of molecular sizes as specified in **Supplementary Data 7** and collected in separate tubes, which subsequently were subjected to digestion and LC-MS/MS analysis.

Supplementary Table 1 - The cell lines used in the study

#	Cell-line	Origin	Sex & Age	Original reference	Source	Description of cell line	Proteomics experiment
1	A-431	Skin	Female, 85	Giard et al., J Natl Cancer Inst 51: 1417 (1973)	DSMZ	Epidermoid carcinoma cell line	PRM
2	A549	Lung	Male, 58	Giard et al., J Natl Cancer Inst 51: 1417 (1973)	DSMZ	Lung carcinoma cell line	PRM, TMT
3	HEK 293	Embryonal kidney	Female	Graham et al., J Gen Virol 36: 59 (1977)	DSMZ	Embryonal kidney cell line, transformed by adenovirus type 5	PRM
4	U-2 OS	Bone	Female, 15	Ponten J., Saksela E. Int. J. Cancer 2: 434 (1967)	LGC/ATCC	Osteosarcoma cell line	PRM, TMT
5	Hep G2	Liver	Male, 15	Aden et al., Nature 282: 615 (1979)	DSMZ	Hepatocellular carcinoma cell line	PRM, TMT
6	HeLa	Cervix	Female, 31	Scherer et al., J Exp Med 97: 695 (1953)	DSMZ	Cervical epithelial adenocarcinoma cell line	PRM
7	RT4	Urinary bladder	Male, 63	Rigby et al., Br J Cancer 24: 746 (1970)	DSMZ/ECACC	Urinary bladder transitional cell carcinoma cell line	PRM, TMT
8	SH-SY5Y	Bone marrow	Female, 4	Biedler et al., Cancer Res 33: 2643 (1973)	DSMZ	Metastatic neuroblastoma, clonal subline of neuroepithelioma cell line SK-N-SH	PRM
9	BJ	Foreskin	Male	Bodnar AG., et al. Science 279: 349-352 (1998)	LGC/ATCC	Adherent fibroblast cell line	TMT
10	CACO-2	Colon	Male, 72	Fogh J., et al. J. Natl. Cancer Inst. 58: 209 (1977)	DSMZ	Colon adenocarcinoma cell line	TMT
11	U-251 MG	Brain	Male	Westermark B., Int J Cancer12: 438 (1973)	BW	Glioblastoma cell line	TMT

Supplementary Table 2 - Summary of genes quantified by proteomics-based methods

Gene	Type of assay	Max	Min	CV% (across	Fold-change	Missing values
ALDH1A2	PRM (ratio-to-standard)	2.33	0.03	173	77.7	2
ANXA1	PRM (ratio-to-standard)	10.38	0.01	74	865.3	0
ANXA3	PRM (ratio-to-standard)	31.75	0.05	107	705.5	0
ASS1	PRM (ratio-to-standard)	4.30	0.01	107	358.0	0
BPGM	PRM (ratio-to-standard)	30.86	0.28	228	110.6	0
CANT1	PRM (ratio-to-standard)	0.70	0.20	45	3.5	0
CAPG	PRM (ratio-to-standard)	15.72	0.02	100	827.1	0
CLPP	PRM (ratio-to-standard)	5.50	1.59	42	3.5	0
COX5B	TMT (reporter intensity)	7.04	2.55	35	2.8	0
CYP51A1	TMT (reporter intensity)	2.48	0.92	40	2.7	0
DDX21	TMT (reporter intensity)	25.57	9.86	37	2.6	0
DECR1	PRM (ratio-to-standard)	15.11	1.20	66	12.6	0
EIF5	TMT (reporter intensity)	19.02	7.58	41	2.5	0
EPS8	PRM (ratio-to-standard)	3.25	0.08	101	39.6	0
ERBB2	PRM (ratio-to-standard)	4.28	0.30	102	14.4	1
HNMT	PRM (ratio-to-standard)	80.43	0.03	187	2513.5	0
KPNA4	TMT (reporter intensity)	3.40	1.78	24	1.9	0
LCP1	PRM (ratio-to-standard)	71.11	0.01	219	6464.7	0
LRRC59	TMT (reporter intensity)	26.10	6.67	55	3.9	0
MCM6	TMT (reporter intensity)	12.67	4.39	29	2.9	0
MEF2D	PRM (ratio-to-standard)	1.28	0.17	81	7.5	0
MSN	PRM (ratio-to-standard)	7.23	0.00	119	7225.0	0
MSN	TMT (reporter intensity)	48.76	8.34	66	5.8	0
NASP	TMT (reporter intensity)	91.58	13.41	62	6.8	0
OSMR	TMT (reporter intensity)	0.52	0.09	52	5.5	0
PAK1	PRM (ratio-to-standard)	3.03	0.13	81	22.9	0
PCYT2	PRM (ratio-to-standard)	30.29	1.64	149	18.4	0
PDIA3	TMT (reporter intensity)	81.23	30.03	38	2.7	0
PIK3R1	PRM (ratio-to-standard)	3.71	0.39	108	9.6	0
PLGRKT	TMT (reporter intensity)	1.24	0.19	57	6.6	0
PMPCA	TMT (reporter intensity)	3.59	1.69	24	2.1	0
PPP1R13L	TMT (reporter intensity)	13.83	3.01	59	4.6	0
PRKCD	PRM (ratio-to-standard)	7.30	0.61	91	11.9	0
RAB3GAP1	TMT (reporter intensity)	10.66	6.07	20	1.8	0
RRBP1	PRM (ratio-to-standard)	16.74	0.68	129	24.8	0
S100A10	TMT (reporter intensity)	8.02	0.88	59	9.2	0
SCAF11	TMT (reporter intensity)	33.12	3.97	117	8.3	0
SELENBP1	PRM (ratio-to-standard)	13.62	0.02	138	567.6	0
SFN	PRM (ratio-to-standard)	8.44	0.15	85	55.2	1
SRC	PRM (ratio-to-standard)	7.22	0.17	99	43.0	0
STOM	PRM (ratio-to-standard)	13.30	0.14	136	97.8	0
STUB1	PRM (ratio-to-standard)	7.30	0.81	68	9.1	0
STX4	TMT (reporter intensity)	3.43	1.79	22	1.9	0
STXBP1	PRM (ratio-to-standard)	0.46	0.02	97	24.4	0
TKT	TMT (reporter intensity)	48.23	10.75	59	4.5	0
TNS1	TMT (reporter intensity)	7.63	0.97	99	7.8	0
TOMM20	TMT (reporter intensity)	0.96	0.58	17	1.7	0
UGDH	PRM (ratio-to-standard)	53.95	1.21	138	44.7	0
VARS	TMT (reporter intensity)	15.41	7.50	25	2.1	0

Supplementary Table 3 - Pearson r and Spearman rho estimates for all Western blot bands and their correlation with MS and RNA data

Orthogonal validation method			**Proteomics validation**					**Transcriptomics validation**			
Gene	Antibody	**Order in Fig 2c**	*Pearson r*	*(p-val)*	*Spearman rho*	*(p-val)*	*MS experiment*	*Pearson r*	*(p-val)*	*Spearman rho*	*(p-val)*
BPGM	HPA016493	1	1	<1E-04	0.58	<1E-04	PRM	0.98	<1E-04	0.58	<1E-04
PCYT2	HPA023034	2	1	<1E-04	0.43	<1E-04	PRM	0.99	<1E-04	0.4	<1E-04
HNMT	HPA035480	3	0.99	<1E-04	0.46	<1E-04	PRM	0.95	0.0004	0.46	0.0004
LCP1	HPA019493	4	0.99	<1E-04	0.87	<1E-04	PRM	0.85	0.0079	0.87	0.0079
PRKCD	HPA001890	5	0.99	<1E-04	0.94	<1E-04	PRM	0.92	0.0013	0.74	0.0013
RRBP1	HPA011924	6	0.99	<1E-04	0.87	<1E-04	PRM	0.73	0.0389	0.85	0.0389
SCAF11	HPA045456	7	0.99	1E-04	0.43	1E-04	TMT	0.94	0.0017	0.39	0.0017
TKT	HPA029481	8	0.99	<1E-04	0.89	<1E-04	TMT	0.87	0.0107	0.36	0.0107
ERBB2	HPA001338	9	0.98	1E-04	0.8	1E-04	PRM	0.74	0.0548	0.53	0.0548
MSN	HPA011135	10	0.98	2E-04	0.96	2E-04	TMT	0.94	0.0015	0.93	0.0015
PIK3R1	HPA001216	11	0.98	<1E-04	0.77	<1E-04	PRM	0.86	0.0059	0.65	0.0059
CAPG	HPA019092	12	0.97	1E-04	0.99	1E-04	PRM	0.98	<1E-04	0.95	<1E-04
DDX21	HPA036593	13	0.96	6E-04	0.89	6E-04	TMT	0.95	0.0012	0.71	0.0012
SFN	HPA011105	14	0.96	6E-04	0.99	6E-04	PRM	0.85	0.0164	0.85	0.0164
TNS1	HPA036089	15	0.96	7E-04	0.8	7E-04	TMT	0.9	0.0051	0.76	0.0051
ASS1	HPA020934	16	0.95	3E-04	0.93	3E-04	PRM	0.72	0.0439	0.85	0.0439
STOM	HPA010961	17	0.95	3E-04	0.88	3E-04	PRM	0.7	0.0537	0.61	0.0537
S100A10	HPA003340	18	0.94	0.0015	0.96	0.0015	TMT	0.51	0.237	0.82	0.237
MEF2D	HPA004807	19	0.93	8E-04	0.67	8E-04	PRM	0.67	0.07	0.76	0.07
CANT1	HPA019639	20	0.91	0.0016	0.87	0.0016	PRM	0.35	0.396	0.19	0.396
PAK1	HPA003565	21	0.91	0.0015	0.98	0.0015	PRM	0.64	0.085	0.76	0.085
PLGRKT	HPA011144	22	0.91	0.0043	0.89	0.0043	TMT	0.82	0.0248	0.82	0.0248
OSMR	HPA017278	23	0.9	0.0062	0.79	0.0062	TMT	0.83	0.0223	0.79	0.0223
EIF5	HPA000867	24	0.89	0.0067	0.68	0.0067	TMT	0.49	0.2636	0.36	0.2636
SRC	HPA030875	25	0.89	0.003	0.8	0.003	PRM	0.73	0.0398	0.56	0.0398
CLPP	HPA010649	26	0.88	0.0042	0.83	0.0042	PRM	0.2	0.6409	-0.12	0.6409
ANXA1	HPA011272	27	0.86	0.0068	0.78	0.0068	PRM	0.91	0.0018	0.92	0.0018
SELENBP1	HPA011731	28	0.86	0.0067	0.87	0.0067	PRM	0.37	0.3672	0.85	0.3672
ANXA3	HPA013398	29	0.84	0.0094	0.93	0.0094	PRM	0.81	0.0149	0.86	0.0149
NASP	HPA028136	30	0.84	0.0169	0.86	0.0169	TMT	0.67	0.0984	0.68	0.0984
LRRC59	HPA030829	31	0.83	0.0217	0.96	0.0217	TMT	0.75	0.0516	0.5	0.0516
TOMM20	HPA011562	32	0.83	0.0213	0.68	0.0213	TMT	0.2	0.6608	-0.18	0.6608
EPS8	HPA003897	33	0.81	0.0138	0.84	0.0138	PRM	0.93	0.0008	0.91	0.0008
CYP51A1	HPA041325	34	0.8	0.0292	0.77	0.0292	TMT	0.3	0.52	0.61	0.52
CLPP	HPA070712	35	0.79	0.02	0.71	0.02	PRM	-0.2	0.6397	-0.55	0.6397
MSN	HPA011227	36	0.76	0.0284	0.79	0.0284	PRM	0.97	0.0001	0.85	0.0001
UGDH	HPA036656	37	0.75	0.0317	0.93	0.0317	PRM	0.89	0.0034	0.98	0.0034
PDIA3	HPA003230	38	0.73	0.0636	0.96	0.0636	TMT	0.04	0.9296	0.43	0.9296
STUB1	HPA041222	39	0.72	0.0439	0.57	0.0439	PRM	0.71	0.0477	0.24	0.0477
COX5B	HPA034517	40	0.66	0.1046	0.75	0.1046	TMT	0.93	0.0027	0.64	0.0027
KPNA4	HPA045500	41	0.63	0.1327	0.43	0.1327	TMT	0.72	0.0656	0.68	0.0656
DECR1	HPA023162	42	0.58	0.131	0.4	0.131	PRM	0.37	0.3699	-0.07	0.3699
STUB1	HPA043531	43	0.58	0.1293	0.55	0.1293	PRM	0.85	0.0072	0.45	0.0072
PPP1R13L	HPA041231	44	0.57	0.1863	0.79	0.1863	TMT	0.78	0.0392	0.79	0.0392
PMPCA	HPA021648	45	0.54	0.2127	0.29	0.2127	TMT	0.86	0.0127	0.75	0.0127
MCM6	HPA004818	46	0.51	0.2392	0.5	0.2392	TMT	0.51	0.2421	0.29	0.2421
VARS	HPA046710	47	0.36	0.4266	0.32	0.4266	TMT	0.53	0.2202	0.32	0.2202
STX4	HPA001330	48	**0.35**	0.4395	0.71	0.4395	TMT	**0.55**	0.2007	0.57	0.2007
HNMT	HPA035481	49	0.23	0.5846	0.43	0.5846	PRM	0.27	0.5122	0.52	0.5122
RAB3GAP1	HPA034494	50	0.19	0.6848	0.07	0.6848	TMT	0.41	0.3654	0.32	0.3654
STOM	HPA011419	51	0.02	0.9557	0	0.9557	PRM	-0.28	0.5005	0	0.5005
STXBP1	HPA008209	52	-0.01	0.9842	-0.19	0.9842	PRM	-0.02	0.9626	0	0.9626
ALDH1A2	HPA010022	53	-0.27	0.6113	-0.39	0.6113	PRM	-0.16	0.7135	-0.65	0.7135

Supplementary Table 4 - The criteria used here for validation of the Human Protein Atlas antibodies and the number of antibodies and genes with enhanced validation using these criteria. If several bands are presented on the Western blot, only the strongest stained band can be validated and this band needs to be clearly separated from the weaker bands (if present)

Validation strategy	Criteria	No ab	No genes
Genetic	At least 25% knock-down in staining on the Western blot for the sample treated with gene-specific siRNA reagent	217	211
Recombinant expression	The antibody must show a strong band on the Western blot for the cell line with recombinant expression and no or faint band in the control.	2,190	1,915
Orthogonal	Correlation in staining on the Western blot for protein target with the antibody-independent method with at least a 5-fold difference in expression levels between two samples. Antibody is also considered validated if the expression levels (trend) in the two cell lines supported the correlation and the size of the band was confirmed either by theoretical size estimate or by the capture MS method described above.	691	644
Independent antibody	Correlation in staining using at least two antibodies with non-overlapping epitopes. See orthogonal for detailed description.	1,922	936
Capture MS	Target protein peptide are present in the same part of the electrophoresis gel as the antibody-stained band in the Western blot	2,888	2,332

Supplementary Table 5 - Success rate per validation pillar

	Ortogonal	Capture MS	Independent	Genetic	Recombinant
Enhanced	711	2888	1922	217	2190
Uncertain	990	762	439	292	62
Not done	4313	2364	3653	5505	3762
Evaluated Abs	1701	3650	2361	509	2252
Success rate (%)	41.8	79.1	81.4	42.6	97.2
Unique Genes	644	2332	936	211	1915

Supplementary Table 6 - QPrESTs used in this assay

QPrEST ID	HPRR	QPrEST ID
UGDH	HPRR3000140	QPrEST21479
STXBP1	HPRR1920071	QPrEST25425
STUB1	HPRR3160243	QPrEST38867
STOM	HPRR1951144	QPrEST23874
SRC	HPRR290019	QPrEST40016
SFN	HPRR1510038	QPrEST24094
SELENBP1	HPRR1370137	QPrEST26348
RRBP1	HPRR1950729	QPrEST38025
PRKCD	HPRR260307	QPrEST25906
PIK3R1	HPRR290105	QPrEST23901
PCYT2	HPRR2501289	QPrEST34825
PAK1	HPRR490019	QPrEST26317
MSN	HPRR1970043	QPrEST23841
MEF2D	HPRR680167	QPrEST26851
LCP1	HPRR2350017	QPrEST23183
HNMT	HPRR2930159	QPrEST24840
ERBB2	HPRR320002	QPrEST22631
ERBB2	HPRR320022	QPrEST40018
EPS8	HPRR620024	QPrEST26221
DECR1	HPRR2540115	QPrEST27322
CLPP	HPRR1440011	QPrEST27335
CAPG	HPRR2370009	QPrEST24357
CANT1	HPRR2390065	QPrEST32926
BPGM	HPRR2100041	QPrEST22779
ASS1	HPRR2470235	QPrEST22452
ANXA3	HPRR2050268	QPrEST23118
ANXA1	HPRR1920070	QPrEST22604
ALDH1A2	HPRR1850063	QPrEST21970
